# Supplementary material for: Resolving the full spectrum of human genome variation using Linked-Reads
Source: Genome Res. 2019 Apr;29(4):635–45. doi: 10.1101/gr.234443.118 (PMC6442396; doi:10.1101/gr.234443.118)
Supplement: Supplemental Material [file supp_gr.234443.118_Supplemental_longranger-2.2.zip › longranger-2.2.tar.bz2!/lr2.2-public/lib/stan/cmdstan/stan/doc/api/html/fwd_2operators_8hpp_source.html]

Stan: src/stan/agrad/fwd/operators.hpp Source File


|  |  |
| --- | --- |
|  | Stan  2.5.0  probability, sampling & optimization |


- Main Page
- Related Pages
- Namespaces
- Classes
- Files

- File List
- File Members

All Classes Namespaces Files Functions Variables Typedefs Enumerations Enumerator Friends Macros Pages

- src
- stan
- agrad
- fwd

operators.hpp

Go to the documentation of this file.

1 #ifndef STAN\_\_AGRAD\_\_FWD\_\_OPERATORS\_HPP

2 #define STAN\_\_AGRAD\_\_FWD\_\_OPERATORS\_HPP

3

4 #include <stan/agrad/fwd/operators/operator\_addition.hpp>

5 #include <stan/agrad/fwd/operators/operator\_division.hpp>

6 #include <stan/agrad/fwd/operators/operator\_equal.hpp>

7 #include <stan/agrad/fwd/operators/operator\_greater\_than.hpp>

8 #include <stan/agrad/fwd/operators/operator\_greater\_than\_or\_equal.hpp>

9 #include <stan/agrad/fwd/operators/operator\_less\_than.hpp>

10 #include <stan/agrad/fwd/operators/operator\_less\_than\_or\_equal.hpp>

11 #include <stan/agrad/fwd/operators/operator\_multiplication.hpp>

12 #include <stan/agrad/fwd/operators/operator\_not\_equal.hpp>

13 #include <stan/agrad/fwd/operators/operator\_subtraction.hpp>

14 #include <stan/agrad/fwd/operators/operator\_unary\_minus.hpp>

15

16 #endif

operator\_greater\_than\_or\_equal.hpp

operator\_addition.hpp

operator\_not\_equal.hpp

operator\_division.hpp

operator\_multiplication.hpp

operator\_equal.hpp

operator\_less\_than.hpp

operator\_unary\_minus.hpp

operator\_greater\_than.hpp

operator\_subtraction.hpp

operator\_less\_than\_or\_equal.hpp


---

[ Stan Home Page ]

*© 2011–2014,
Stan Development Team.*
